# Supplementary material for: Low-level factors increase gaze-guidance under cognitive load: A comparison of image-salience and semantic-salience models
Source: PLoS One. 2022 Nov 28;17(11):e0277691. doi: 10.1371/journal.pone.0277691 (PMC9704686; doi:10.1371/journal.pone.0277691)
Supplement: Attachment — Submitted filename: SceneProcessing_SalienceSemantics - PLOS_Response2.docx [file pone.0277691.s004.docx]

Dear Editor and Reviewer,

Thank you for your comments on our Manuscript titled “Low-Level Factors Increase Gaze-Guidance Under Cognitive Load: A Comparison of Image-Salience and Semantic-Salience Models”.

We have revised the manuscript to address the final review points. The editor and reviewer comments are in italics below, followed by our responses. We have highlighted our revisions to the manuscript under our “Revised Manuscript with Track Changes” submission. We think the paper is much improved thanks to these revisions and hope that you can accept the revised manuscript for publication in PLOS ONE.

Sincerely,
Kerri Walter and Peter Bex

Journal Requirements:

Please review your reference list to ensure that it is complete and correct. If you have cited papers that have been retracted, please include the rationale for doing so in the manuscript text, or remove these references and replace them with relevant current references. Any changes to the reference list should be mentioned in the rebuttal letter that accompanies your revised manuscript. If you need to cite a retracted article, indicate the article’s retracted status in the References list and also include a citation and full reference for the retraction notice.

*The manuscript has been checked and all references are current and relevant.*

Additional Editor Comments (if provided):

Please, we need that you consider to rephrase the comment from reviewer 1 on page 6.

*Reviewer 1’s comment has been addressed in the manuscript.*

*Additionally, after uploading figures to PACE, figures with individual subject lines (fig5-fig7) became blurred by the thinness and lightness of the individual subject traces. We have edited the lines so that they are darker and clearer after being uploaded to PACE.*

Reviewers' comments:

Reviewer #1: The authors have addressed all of my comments with one exception. I feel that their characterization of the relation between the present study and Walter & Bex (2021) was a bit vague. On p. 6, they wrote that "In the present study, we utilize the same paradigm...". If I understand correctly, they used the same data. In other words, in the present study, the authors reanalyzed the data that was published in the previous study. The new analyses are sufficiently different from those of the previous study, so I'm not suggesting that this is a problem. My point is simply that it should be clear to the reader that the data are the same.

Other than this one minor point, I think that the paper is suitable for publication. The authors did an excellent job responding to my concerns and the revised version will make an interesting contribution to the literature.

*It has been emphasized in the introduction of the present study (pg. 5) that the data used in the present analysis are that of the study from Walter & Bex (2021)*.
